# The Antioxidant Capacities of Natural Products 2019

**DOI:** 10.3390/molecules25235676

**Published:** 2020-12-01

**Authors:** Susana M. Cardoso, Alessia Fassio

**Affiliations:** 1 LAQV-REQUIMTE, Department of Chemistry, University of Aveiro, 3810-193 Aveiro, Portugal; 2 Department of Pharmacy, Health and Nutritional Sciences, University of Calabria, 87036 Rende, Italy

The search for new natural antioxidants is a growing area of research due to the broad spectrum of their biological properties, which are associated with the prevention of chronic diseases that originate in oxidative stress. In this context, the fact that they promote a health capacity is noteworthy and has determined a marked trend towards the supplementation of food products with natural antioxidants. This Special Issue of *Molecules* contains a collection of 28 research works and 4 reviews, overall covering distinct aspects related to natural antioxidants.

Several authors highlighted the relevance of specific structural features of natural compounds in their antioxidant capacities. In this context, the comparison made by Ouyang et al. [1] between galangin and 3,5,7-trihydroxychromone (characterized by the presence or absence of a null B-ring) allowed the authors to elucidate that, regardless of it not affecting the antioxidant pathways, the presence of the null B-ring in flavonols improves the antioxidant levels, since the π–π conjugation can provide more resonance forms and bonding sites. Moreover, Li and coworkers [2] showed that the presence of 3,8″-dimerization on flavonoids could enhance the antioxidant capacity through the electron-transfer pathway, possibly by allowing a partial π–π conjugation. Relevance was also given to the influence of substituents on the modulation of the antioxidant activity. In particular, when comparing the antioxidant potential of scutellarein and scutellarin (scutellarein-7-O-glucuronide), Liu and coworkers [3] revealed that the glucuronidation of pyrogallol-type phytophenol antioxidants caused a dual effect by decreasing the antiradical potential towards several radicals such as 1,1-diphenyl-2-picrylhydrazyl, 2,2′-azino-bis(3-ethylbenzothiazoline-6-sulfonic acid), 2-phenyl-4,4,5,5-tetramethylimidazoline-1-oxyl 3-oxide radicals and the ability for radical adduct formation, and by in turn enhancing the Fe^2+^-chelating potentials. Also worth noting, the work performed by Liang et al. [4] with the two chalcones echinatin and licochalcone A allowed them to conclude that the 1,1-dimethyl-2-propenyl substituent increased its antioxidant potential, which in aqueous solutions may occur through an electron transfer and proton transfer mechanism and, in addition, in an alcoholic solution through hydrogen atom transfer preferentially at 4-OH.

Another research direction in focus in this special edition aims to elucidate the mechanisms of protection of natural compounds and/or to explore new sources of health-promoting compounds. Among the published works, phenolic compounds or derivatives were spotlit because of their antioxidant abilities, albeit other potential bioactivities such as cytoprotective, antiproliferative and cytotoxic ones were also investigated. Notably, Das and coworkers [5] elucidated multiple protective mechanisms involving the phenolic (catecholic) diterpene carnosic acid against cadmium-provoked nephrotoxicity. As for the natural sources rich in phenolic compounds, these included distinct parts of plants (the root, xylem, phloem, petiole, leaves and bud of *Boehmeria nivea* L. [6]), with emphasis on medicinal plants (aerial parts of *Salvia africana*, *Salvia officinalis ‘Icterina’* and *Salvia mexicana* [7], leaves of *Sorbus domestica* [8], leaves of *Cotoneaster zabelii*, *Cotoneaster bullatus* and *Cotoneaster integerrimus* [9], leaves of *Mahonia bealei* (Fort.) Carr [10], leaves of *Vaccinium vitis-idaea* L. [11], flowers of *Astragalus membranaceus* var. mongholicus [12], rhizomes of *Reynoutria japonica*, *Reynoutria sachalinensis* and *Reynoutria* x bohemica [13], fruits of *Lycium barbarum* L. [14]), the pericarp of *Dimocarpus longan* Lour. [15], the resin of the tree species Croton lechleri (Müll. Arg) [16] and a herbal standardized product containing an extract of persimmon leaves [17]. In parallel, nonphenolic compounds, including monoterpes and their derivatives from three mentha species [18], alkaloids from *Uncaria tomentosa* [19], polysaccharides from white ginseng [20] and protein hydrolysates from walnut meal [21], were also demonstrated to hold the potential to be used as natural antioxidants and/or to fight oxidative stress related disorders.

Attention was also given to the impact of distinct factors on the levels of relevant natural compounds, including bioactive components of plants/vegetables. For example, the work of Liu et al. [22] demonstrated that sunny hours and temperature were the main drivers affecting the accumulation of *Cyclocarya paliurus* phenolics and their antioxidant properties. In addition, Guan and coworkers [23] concluded that the combination of methyl jasmonate treatment with wounding stress could stimulate phenolic accumulation in broccoli. Moreover, Pereira et al. [24] described the variable effects of commercial biostimulants and irrigation regimes on the chemical composition and bioactive properties of two spinach genotypes, alerting one to the need for further research in order to make solid conclusions on the effects of the use of biostimulants under water stress conditions.

The application of natural compounds as ingredients in novel products to emphasize specific characteristics or new strategies to improve the quality and antioxidant properties of health-promoting natural products/bioactive compounds were also addressed by some contributing authors. This included the formulation of a novel fermented glutinous rice product with the supplementation of Fu brick tea (i.e., a beverage processed through the postfermentation of *Camellia sinensis* L.) to increase its sensorial features, the levels of phenolics, and its antioxidant and DNA protective activities [25]; the supplementation of pasta flour with salmon fish powder to manipulate the glycaemic index, protein digestibility, release of phenolic compounds and antioxidant capacity of the digested pasta [26]; and the use of cold saponification on commercial natural soaps manufactured from plant oils and additives in order to retain unsaponified fatty acids, phenolic compounds and antioxidant activities in the final products [27]. In addition, Wu et al. [28] explored a new strategy for ameliorating the stability and enhancing the stability, solubility and safety of resveratrol through the preparation of novel resveratrol transfersomes.

Campos et al. [29] provided an overview on the valorization of fruit byproducts from food-processing industries in order to overcome a global problem, highlighting the application of sustainable and green methodologies for the conversion of fruit waste into high-value products with a significant biological activity. Santos and Silva [30] summarized different prenylation patterns of natural and synthetic flavonoids that have been focused on in the past two decades, aiming at the elucidation of structure-antioxidant activity relationships and the development of efficient routes for the synthesis of natural derivatives. All of the studies published over the last decade on the relationship between moderate alcohol consumption and coronary heart disease were revised by Castaldo et al. [31], who summarized the various red wine components and the putative mechanisms that influence their activity. Cione et al. [32] described four polyphenols used as nutritional supplements: quercetin, epigallocatechin gallate, curcumin and resveratrol, summarizing the current knowledge about them, ranging from dietary sources to human microRNA modulation.
